# Urologists’ and general practitioners’ knowledge, beliefs and practice relevant for opportunistic prostate cancer screening: a PRISMA-compliant systematic review

**DOI:** 10.3389/fmed.2024.1283654

**Published:** 2024-02-16

**Authors:** María Estevan-Ortega, Cristina de la Encarnación Castellano, Alberto Mendiola-López, Lucy A. Parker, Juan Pablo Caballero-Romeu, Blanca Lumbreras

**Affiliations:** ^1^Pharmacy Faculty, University Miguel Hernández de Elche, Alicante, Spain; ^2^Department of Urology, University General Hospital of Alicante, Alicante, Spain; ^3^Department of Public Health, University Miguel Hernández de Elche, Alicante, Spain; ^4^CIBER de Epidemiología y Salud Pública (CIBERESP), Madrid, Spain; ^5^Alicante Institute for Health and Biomedical Research (ISABIAL), Alicante, Spain

**Keywords:** prostate cancer, screening, knowledge, urologists, general practitioner (GP)

## Abstract

**Background:**

Recent guidelines on opportunistic prostate cancer screening conclude that the decision to screen with prostate-specific antigen should be made by each patient individually together with the clinician. However, there is evidence of a lack of clinicians’ awareness of prostate cancer screening. This study sought to assess the recent evidence of clinicians’ knowledge, beliefs, and practice regarding opportunistic prostate cancer screening comparing urologists and generals practitioners.

**Methods:**

A systematic search was conducted in 3 online databases: MEDLINE, Web of Science and EMBASE (from January 1, 2015, to January 9th, 2023). Studies that explored clinicians’ knowledge, beliefs, and practices regarding opportunistic prostate cancer screening were included. Studies were assessed for quality reporting according to the Strengthening the Reporting of Observational studies in Epidemiology guidelines.

**Results:**

A total of 14 studies met the inclusion criteria: ten studies included primary care health professionals, three studies included urologists, and one study included both. Studies involving general practitioners showed a generally low level of awareness of the recommended uses of the test, and urologists showed a greater knowledge of clinical practice guidelines. General practitioners’ opinion of prostate-specific antigen was generally unfavourable in contrast to urologists’ who were more likely to be proactive in ordering the test. Less than half of the included studies evaluated shared-decision making in practice and 50% of clinicians surveyed implemented it.

**Conclusion:**

General practitioners had less knowledge of prostate cancer risk factors and clinical practice guidelines in the use of PSA than urologists, which makes them less likely to follow available recommendations. A need to carry out education interventions with trusted resources based on the available evidence and the current guidelines was identified.

## 1 Introduction

Screening for prostate cancer (PCa) using prostate-specific antigen (PSA) seeks to detect PCa at an early stage to reduce disease-specific mortality (1). Data from the European Randomised study of Screening for Prostate Cancer (ERSPC) (2), which included 182,160 men, showed that PSA screening significantly reduced prostate cancer–specific mortality by 20% at 16 years of follow-up. Although the Prostate, Lung, Colorectal and Ovarian (PLCO) Cancer Screening trial failed to show a reduction in PCa–specific mortality (3), a recent modelling analysis on the data from ERSPCA and PLCO trials showed a reduction of approximately 25–32% in PCa mortality (4). Nevertheless, there are controversies regarding its use as a screening test, as it is also associated with false-positive results and a high frequency of overdiagnosis (5).

Weighing the benefits and harms of PSA, the U.S. Preventive Services Task Force (USPSTF) updated its recommendations in 2017. They stated that opportunistic screening may be useful for men aged 55–69 years, but the decision to screen should be made by each patient individually together with the clinician after the patient has understood the benefits and risks of screening (6). The European Association of Urology (EAU) (7) updated their recommendations in 2015 and more recently in 2021. They stated that clinicians should offer an individualized early detection strategy to inform patients aged over 50 years old with a good functional status and a life expectancy of at least 10–15 years, to African American patients and patients with a family history of PCa aged over 45 years and to men carrying BRCA2 mutations over 40 years old. In addition, they have recently published recommendations for the use of PSA testing as part of a risk-adapted strategy aimed at tackling the present situation in most countries in the European Union (EU). Nevertheless, PSA testing is being prescribed for men over 50 as well as those over 70 in a yet unorganized or on-request service (8), which results in a high rate or false positive results and overdiagnosis. Recently, the European Union published Europe’s Beating Cancer Plan (9), which proposes the introduction of PCa screening with prostate-specific antigen (PSA) testing for men up to 70 years old in combination with additional magnetic resonance imaging (MRI) scanning as a follow-up test.

Healthcare providers play a crucial role in influencing PCa screening uptake among men, providing them with essential information on related risks, potential benefits and uncertainties (10). However, discrepancies in the approach to PSA testing and adherence to PCa screening guidelines are reported between general practitioners (GPs) and urologists. GPs, compared to urologists, may: perceive the PSA test as less useful, show a less proactive approach in informing men about PSA, and exhibit less familiarity with screening guidelines (11). Knowledge gaps among GPs have been found (12), and this point is relevant since the knowledge and attitudes of primary healthcare providers may influence their approach to PCa screening and their implementation of SDM. In this sense, a study in the United States revealed suboptimal practice of SDM among some GPs involved in PCa screening with PSA (13). Controversy surrounding PCa screening and the recent updating of the available guidelines could influence lack of knowledge among GPs and thus, their uneven handling of PSA testing.

Previous research showed a significant decline in the use of PSA screening among men aged 50 and above following the release of the 2012 US Preventive Services Task Force (USPSTF) guidelines (14). Despite this overall reduction, PSA screening continues to be performed at levels that seemingly contradict the USPSTF recommendation. This discrepancy raises questions about the potential influence of the guidelines on the clinicians’ practice (15).

Although there is evidence on clinicians’ knowledge, beliefs and practices before the updating of the available guidelines (16), there has not been an analysis of this information since the last updates of the European Association of Urology guidelines (from 2015 onwards) and USPSTF (after 2017). Moreover, no evidence has been published regarding clinicians’ familiarity with the guideline statements and their opinions about them since the recent updates for both GPs and urologists.

This study, therefore, aims to fill this gap by comparing the recent evidence of GPs and urologists’ (population) about: a) knowledge, b) beliefs, and c) practice (outcomes) regarding opportunistic PCa screening with PSA determination (intervention/exposure). This knowledge will be useful for designing targeted strategies to provide education for clinicians following the recent European Union Cancer Plan.

## 2 Methods

This review was reported according to the PRISMA statement (Preferred Reporting Items for Systematic Reviews and MetaAnalyses) (17) (protocol in Supplementary Table S1).

### 2.1 Inclusion and exclusion criteria

The population, intervention, comparator and outcomes (PICO) framework (18) was used to define the eligibility criteria. Studies reporting original research that met the following criteria were included:

- Population— Clinicians: general practitioners and urologists.
- Intervention/exposure— opportunistic screening of prostate cancer based on PSA test.
- Comparison—none.
- Outcomes—clinicians’ knowledge (urologists and GPs), beliefs and practice regarding opportunistic prostate cancer screening with PSA determination.

Observational studies published in English or Spanish that assessed clinicians’ knowledge, beliefs and practices regarding opportunistic PCa screening with PSA determination were included. We restricted to those published after 2015.

### 2.2 Search strategy

We searched the following databases by 9th of January, 2023, MEDLINE (through PubMed), Web of Science and EMBASE using terms referring to the population (health professionals), intervention (knowledge, beliefs and practice) and outcome (screening request) as descriptors or keywords.

Searches for descriptors were carried out in English and combined by Boolean operators (OR and AND) in four blocks: clinicians; prostate cancer; screening; knowledge, beliefs, practices. The descriptors in each block were combined by the Boolean operator OR. The combination between the blocks was done using the AND operator. Forward and backward citation searching was performed on included papers. The detailed search strategy is outlined in Supplementary Table S2.

To assess risk of bias due to missing results, we also checked for publication in other languages and there were no studies that met the eligibility criteria.

### 2.3 Study selection

All records retrieved from the search were imported into EndNote, deduplicated and then imported into Rayyan for screening (19). Two reviewers (MEO and BL) independently screened each reference title and abstract (if available) for relevance to this review and eliminated duplicates. This first screening excluded editorials, letters to the editor, systematic reviews, study protocols and any study that did not include original data.

The full article of the selected studies in the first screening was then reviewed. The second round of screening involved two reviewers (MEO and BL) independently and was based on the application of the selection criteria. Any discrepancies in the two screenings between the two reviewers were discussed with two other reviewers (CEC, AM, two urologists with expertise in the field). Study investigators or published studies were not contacted for more additional information.

### 2.4 Data extraction, variables included and quality assessment

The following data from each study were obtained: country and date of publication, objective, study design, study population (inclusion and exclusion criteria, sample size, and classified into GPs or urologists), sociodemographic characteristics of the population included (sex, age), procedure, main results (knowledge, beliefs and practice), conclusions and limitations.

Studies were assessed for reporting quality according to the STrengthening the Reporting of OBservational studies in Epidemiology (STROBE) guidelines (20).

For both the extraction of the main variables and the quality assessment, three of the authors (MEO, CEC and AML) reviewed the studies independently, and disagreements were resolved by discussion and consensus with other reviewer (BL). Cohen’s kappa coefficient between the reviewers was 1.00.

### 2.5 Data synthesis and analysis

Data were collated and synthesised using narrative and descriptive summaries. No attempt at meta-analysis was made given the heterogeneity in target population, study design and outcome measures across included studies. To improve conceptual clarity and comprehensiveness, two independent researchers (BL and MEO) synthesized for each report the knowledge, attitudes, beliefs and practice (and their analysis) for the different population (i.e., GPs, urologists).

## 3 Results

### 3.1 Literature search

**Figure 1:**
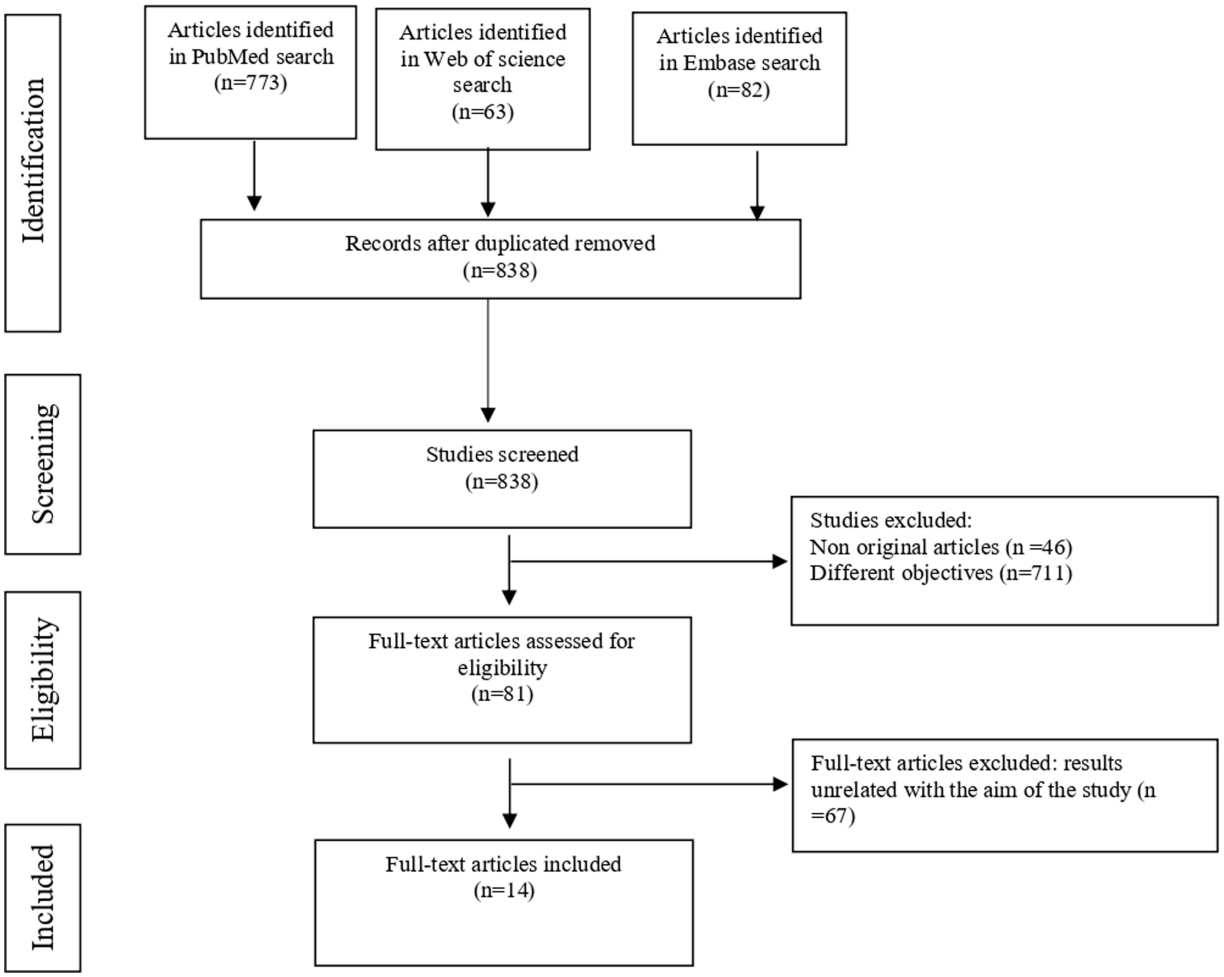
Flowchart describing study selection and excluded studies, according to PRISMA recommendations.

The systematic searches yielded 918 potentially relevant citations, of which 80 were duplicates. A systematic screening process was used (Figure 1) to screen titles, abstracts, and full-text publications, resulting in 81 eligible studies. The reason for the exclusion of full texts was mainly that the results were unrelated to the aim of the study. Finally, 14 studies met the inclusion criteria (11, 21–33).

### 3.2 Evaluation of quality reporting

The median compliance with the 22 criteria applied was 17.5 (IQR 16.8–20) (Supplementary Tables S3, S4).

All studies described the criteria related to the description of the background (item 2), key elements of the study design (item 4), selection of participants (item 6), quantitative variables (item 11), description of the main results (items 13–15), and discussion of the limitations (item 19) and interpretation of results (item 19). However, criteria related to sensitivity analysis (item 17), explanation of how the study size was arrived (item 10) and how quantitative variables were handled in the analyses, were only described in 4 (28.6%), 8 (57.1%) and 8 (57.1%) studies, respectively.

There were three studies (14.3%) below the first quartile (11, 23, 30) and four studies (28.6%) between the median and the first quartile (11, 24, 31, 32). Miller et al’s study (23) did not include relevant aspects such as a detailed description of the setting, thereby limiting the external validity of the results. Furthermore, the study failed to adequately articulate the outcomes and exposure variables, and the methodology for determining the study size was not clearly outlined, potentially introducing biases. Benedict Moa et al.’s study (30) did not provide an explanation for the origin of various variables. In the case of Kappen S et al.’s study (11), the research objectives were not explicitly stated, and there was a lack of effort to address potential sources of bias. Additionally, the study did not detail how the study size was determined and failed to provide a comprehensive description of the statistical analysis.

Concordance among the reviewers was 95.4%.

### 3.3 Characteristics of identified studies

All articles were cross-sectional, and 7 of them (50%) were published between 2015 and 2017. Most of them were carried out in the USA (5, 35.7%) (21, 23, 25, 29, 31), 2 (14.3%) in Spain (24, 26), and the rest in countries such as Malaysia (22), Saudi Arabia (27), Netherlands (28), South Africa (30), Caribbean (32), Switzerland (33), and Germany (11) (Table 1).

**Table 1:** Description of the main characteristics of the 14 studies included in the review.

Author, year	Country	Objective	Population	Age	Sex	Sample size	Characteristics of the questionnaire
General practitioners (GPs) and other primary care professionals							
Elstad, 2015 (21)	United States	GPs’ perceptions of the harms/benefits of 2 screening techniques (colonoscopy/PSA).	GPs	Mean: 45 years	Men: 62%	126 (80% response rate)	Paper survey that included 2 vignettes with a hypothetical patient.
Malik, 2016 (22)	Malaysia	GP’s awareness and practice of PCa screening.	GPs	Mean 48.3 years	Men: 65.3%	196 (65% response rate)	Via postal mail and clinic visits: a questionnaire adapted from a previous survey developed by Drummond et al., content and face validated and analysed in a pilot study.
Miller, 2016 (23)	United States	Impact of the U.S. Preventive Services Task Force (USPSTF) guideline of PCa screening on the GPs’ attitudes and knowledge.	GPs	Mean: 52.1 years	Men: 49%	73 (response rate 21%)	Online survey: a 25-item questionnaire
Panach-Navarrete, 2016 (24)	Spain	GPs’ use of PSA by GPs in relation to patient age, the value of PCa screening, and subjective beliefs about its usefulness.	GPs	Mean: women 50,2 years; men 51,8 years	Men: 38,8%	103	Paper form survey: a 15-item questionnaire
Hall, 2017 (25)	United States	Differences in knowledge between GPs and internal medicine physicians.	GPs and internal medicine physicians	NA	Males: 72%	1,003: 480 GPs and 523 internal medicine physicians (response rate 70.5)	DocStyles Web-based survey developed by Porter Novelli with guidance provided by federal public health agencies and other non-profit and for-profit clients.
Giménez, 2018 (26)	Spain	Usual practice and perception of GPs and laboratory physicians on PSA screening for PCa.	GPs and laboratory physicians	Mean 43 years	Men: 36%	341: 114 GPs (response rate 70%) + 227 laboratory physicians (response rate 8.8%).	Via online and in person: The version for GPs contained 40 variables and for laboratory physicians, 36 variables (values from 1 to 10). An initial technical validation (comprehension and relevance) was carried out with experts. A pilot test was carried out with 30 GPs.
Nassir, 2019 (27)	Saudi Arabia	Knowledge among clinicians in the management of the most common urological problems in Saudi Arabia.	GPs and internal medicine clinicians	NA	Men: 57%	112 (75.7% response rate).	Paper form survey: a 21-item questionnaire.
Kappen, 2020 (28)	Netherlands	GPs’ approaches, attitudes and knowledge about PSA use according to the NHG (Dutch clinical guideline).	GPs	Mean: 54 years	Men: 70.9%	88 (response rate 49.2%)	In person questionnaire: a translated and adapted questionnaire with 31 items. Pretest were carried out by urologists and GPs to assess acceptance, comprehensibility, ease of use, feasibility and validity.
Shungu, 2022 (29)	United States	GPs’ approach PCa screening and specifically in black men.	GPs	20–39 years: 420 (35.4%) 40–59 years: 549 (46.2) >60 years 219 (18.4)	Men: 40.3%	1,192 (response rate 32.5%)	Online survey: Council of Academic Family Medicine’s members were invited to propose survey questions. Educational Research Alliance Research Mentor helped refine questions. The final draft was modified following pilot-testing.
Benedict, 2023 (30)	Free State, South Africa.	Primary health care providers’ knowledge, attitudes and practice regarding PCa screening.	Primary health care provider workers: doctors, nurses, clinical associates and community health workers	Mean: 38 years (range 22–77 years)	Men: 23.7%	548 (response rate 71.8%)	A self-administered questionnaire adapted from previous similar surveys and validated by experts specializing in urology, public health, health education and behavioural sciences. It was reviewed and approved by a Health Sciences Faculty evaluation committee and pretested on 22 participants.
Urologists							
Rudichuk, 2017 (31)	United States	Urologists’ knowledge and use of family history to determine recommendations for PCa screening and treatment.	Chicago Urological Society urologists.	27.6% of participants aged 31–40 years	Men: 86,2%	87 (response rate 60).	Paper form survey: a 33-item questionnaire developed with input from genetic counselors, a PhD urologist, and a PhD statistician.
Persaud, 2018 (32)	Caribbean	Caribbean urologists’ attitudes, beliefs and practices regarding PSA testing.	Urologists from the Caribbean Urological Association	Mean: 49.7 years	NA	30 (response rate 75%).	Online survey: a standardised questionnaire designed *ad-hoc*.
Scherer, 2023 (33)	Switzerland	Internists’ and urologists’ personal PSA screening activity as an indicator of their attitude towards PSA screening.	Members of the Swiss Society of Urology and the Swiss Society of General Internal Medicine	Mean: 54.4 years (sd 11.5)	Men: 72.5%	1.083 (response rate 14%).	A 10-item survey consisted of demographic questions about age, sex, medical specialty and work setting (in English, German and French)
GPs and urologists							
Kappen, 2019 (11)	Germany	Differences between GPs and urologists in PSA testing and use of guidelines.	GPs and urologists	GPs: median: 54.0 years Urologists: median: 51.5 years	Men: GPs 87.8% and urologists 100%.	65 (41 GPs and 14 urologists).	Online survey: a questionnaire with 43 topic and four case sceneries. It was tested by three GPs and 1 urologist.

In 10 of the articles (71.4%) (21–30), the study population comprised general practitioners (GPs) and other primary care health professionals [laboratory clinicians (26), nurses, clinical associates and community health workers (30)]; in 3 articles (21.4%) (31–33), urologists were included, and in one, urologists and GPs (11). The mean age of the participants was 49.5 years. The mean sample size was 285, ranging between 30 and 1,192.

### 3.4 Procedural characteristics of the included studies

Data collection was carried out by questionnaire or survey: 9 studies (64.2%) (11, 22, 23, 25, 26, 29, 30, 32, 33) requested the information by e-mail, online, etc., and 3 of them also solicited information directly from participants in person (22, 26, 30). The remaining articles (21, 24, 27, 28) included in person participation in the questionnaires/surveys. The questionnaires/surveys used were mostly designed *ad hoc* for the study, and 3 of the articles (21.4%) (22, 28, 30) included previously used questionnaires. However, only three of the studies indicated that the questionnaire was previously validated (22, 26, 30), and others were previously tested by clinicians (11, 28, 29) (Table 1).

### 3.5 Clinicians’ knowledge, beliefs and use in practice on PCa screening with PSA

The results are described below according to the main topics covered in the studies and in accordance with the clinical specialty (Table 2).

**Table 2:** Analysis of the main results obtained from the 14 studies included in the review according to the following categories: knowledge, beliefs and practice.

Author, year	Results		
	Knowledge	Beliefs	Practice
GPs and other primary care professionals			
Elstad, 2015 (21)		*Benefits and risks* Clinicians listed more harms than benefits of PSA testing. Benefits most frequently mentioned: Early detection and treatment: 72% Psychological effects (e.g., peace of mind): 37% Harms most frequently mentioned: Unnecessary treatment: 56% Psychological effects (e.g., anxiety): 53% Follow-up: 47%	
Malik, 2016 (22)	*Risk factors/characteristics of the test* 56-64% overestimated the positive predictive value of PSA. Risk factors: 82.7% knew that having a relative with PCa and 97.4% that being >50 years old was a risk of PCa. 31.1% knew that having a first-degree relative with breast cancer increased the risk.	*Usefulness of the test* 51.5% believed that healthy men aged 50 years should be tested for PSA annually or less. 22.4% thought that a PSA test should be performed only when a man with risk factors develops lower urinary tract symptoms. 89.8% considered undergoing a PSA test themselves.	*Use of PSA test in practice* 49.5% usually screened asymptomatic patients and 94.9% used PSA for screening. 76% informed patients that their PSA was being checked. *SDM* 61.2% discussed the implication of an abnormal result. 20.4% discussed treatment for PCa before PSA testing.
Miller, 2016 (23)	*Guidelines* 30% were very familiar with the USPSTF guidelines (90% from somewhat to very familiar).	*Benefits and risks* 71% agreed that PSA testing may impart more harm than benefit to patient. *Usefulness of the test* 22% were concerned that not recommending PSA screening could lead to future litigation.	*SDM* 24% felt very comfortable discussing the risks and benefits with patients. 75% claimed to have changed their PSA screening routine based on the guidelines. 59% engaged patients in a shared decision making. 64% support patients having a PSA test if they had weighed the benefits and risks.
Panach-Navarrete, 2016 (24)	*Risk factors/characteristics of the test* 83.5% claimed to have sufficient knowledge about PSA characteristics.	*Usefulness of the test* 64.1% questioned the true usefulness of PSA test. 29.1% believed PSA test is not very useful and 66% quite useful in diagnosing PCa.	*Use of PSA test in practice* 53.4% would not order their first PSA until their 50s, and up to 49% order their first PSA until their 80s. 53.9% would order a PSA per year in a 65-year-old man with no treatment and with a last PSA test of 3 ng/mL one year ago.
Hall, 2017 (25)		*Usefulness of the test* 74% felt that men with risk factors should be tested annually for PSA and 37% felt it should be done in patients >50 years even if they were asymptomatic. 40% agreed that the test has adequate characteristics to be considered a screening test. 75% did not agree with the age range at which the test should be done.	*Use of PSA test in practice* 60% only recommended the test considering individual risk, 25% routinely did it, and 14% did not offer it. The recommendation of the test was related to years of practice, patient request and belief in the efficacy of the test. GPs had greater odds (adjusted OR = 1.54, 95%CI 1.15, 2.07) of considering patient request for the PSA test than internal medicine providers.
Giménez, 2018 (26)	*Guidelines* The professionals’ knowledge of the clinical practice guidelines did not score 5 points on a scale of 1 to 10. Laboratory professionals gave the highest score to the European Guideline on Tumour Markers (4.9 ± 2.8 points). GPs mostly followed (3.6 ± 2.7 points), the recommendations of the Spanish Society of Family and Community Medicine.	*Usefulness of the test* GPs (5 ± 2.4 points) and laboratory clinicians (5.7 ± 2.4 points) showed uncertainty when ordering PSA as a screening test. The main concerns were delayed diagnosis of PCa (GPs: 5.7 ± 2.6 points and laboratory clinicians 6.5 ± 2.3 points) and overdiagnosis and overtreatment of PCa (GPs: 5.8 ± 2.5 points and laboratory clinicians 7.3 ± 2.1 points).	*Use of PSA test in practice* GPs (8.9 ± 1.7 points) and laboratory clinicians (8.3 ± 2 points) showed interest in assessing the prostate clinic before requesting PSA test. GPs explained the consequences about a high PSA test (8.3 ± 2.0 points); they thought that the most suitable age range for PSA screening was 60 years and older (6.4 ± 2.8 points) and the most appropriate time interval for requesting a new PSA test was annually (6.6 ± 2.9 points). Laboratory clinicians showed concerns about false-positive PSA in cancer screening (6.7 ± 2.2 points). Laboratory clinicians (6 ± 2.1 points) showed more interest in asking a PSA test as opportunistic screening than GPs (4.9 ± 2.9 points) and as populational screening (5.5 ± 1.5) points vs. (3.3 ± 2.5 points).
Nassir, 2019 (27)			*Use of PSA test in practice* 2.8% of respondents did not routinely recommend PSA. 58.2% of respondents recommended PSA to >80 years (especially residents).
Kappen, 2020 (29)	*Guidelines* 95% reported to have at least read the Dutch guideline for GPs. 50% reported to be aware of the guideline content.	*Usefulness of the test* 39.1% declared not afraid and 47.1% neutral about their concern on missing PCa in a patient. 28.7% thought screening for PCa is important and 43.7% declared it was neutral.	*SDM* 46% would offer detailed advice before ordering a PSA test to an asymptomatic man who asked for it. *Use of PSA test in practice* 25% would not recommend the test to their family members and 38% would probably not.
Shungu, 2022 (29)	*Guidelines* 70.1% correctly identified the most recent USPSTF PCa screening recommendation.		*Use of PSA test in practice* 87% used the USPSTF as their primary source of information. 69.4% screened with PSA alone and 24.8% with PSA and digital rectal exam. *SDM* 29.2% informed black men aged 55–69 years of their increased risk of developing PCa and 12.1% only if the patient introduces the topic. They engaged in shared decision-making for PCa screening in about 50.4% of eligible white men vs. 54.8% black men.
Benedict, 2023 (30)	*Risk factors/characteristics of the test* 64.8% had poor knowledge about PCa screening, 30.1% had moderate knowledge and 5.1% had good knowledge. Medical officers or GPs, more state-employed participants, participants with prior working experience in urology, participants involved with the training of medical students, and those following PCa screening guidelines in their practice, had better knowledge.	*Usefulness of the test* 58.6% had a neutral attitude towards PCa screening, 40.7% had a negative attitude and 0.7% had a positive attitude. Female participants and professional nurses and community health workers were moer uncomfortable with practice: those with 1–5 years’ working experience had a positive attitude.	*SDM* 40.0% had poor practice regarding PCa screening and SDM, 35.8% had fair practice and 24.3% had good practice. Female participants and participants without additional postgraduate qualifications had poor practice; medical officers or GPs had good practice, state-employed participants, participants with 1–5 years’ working experience, participants involved with training of medical students, and those following PCa screening guidelines in their practice, had good practice.
Urologists			
Rudichuk, 2017 (31)	*Risk factors/characteristics of the test* 83% linked family history and race (92.9%) to an increased risk of PCa.		*Use of PSA test in practice* Respondents chose the recommendation to start PSA testing earlier (<55 years) if patients have a family history of PCa. 87.4% reviewed family history of PCa when considering screening options.
Persaud, 2018 (32)		*Benefits and risks* 66.7% believed thar PSA screening had positively impacted survival in their patient population. *Usefulness of the test* 76.7% supported PSA screening in the asymptomatic Afro-Caribbean men. 35.7% of urologists felt that the patient understood the discussion on screening. 22% believed the international screening guidelines were applicable to the Caribbean and 63% believed that a multinational committee should lead Caribbean screening guidelines.	*SDM* 50% always fully discussed pros and contra PSA screening with patients.
Scherer, 2023 (33)			*Use of PSA test in practice* Male urologists >50 years of age screened themselves more often than male internists >50 years of age (89% vs. 70%, *p* < 0.05). Urologists reported recommending screening statistically significantly more often than internists to their brother, father or partner regardless of their sex (men: 38.1% vs. 18.5%; *p* < 0.05; women: 81.8% vs. 32.2%; *p* < 0.05).
GPs and urologists			
Kappen, 2019 (11)			*Use of PSA test in practice* 65.9% GPs had a standard procedure regarding PSA testing vs. 85.7% urologists. 100% urologists inquired if the patient wishes to do a PSA test (85.7% orally). 24.4% GPs did not ask the patient if he wishes to do a PSA test (73.2% orally). 75.6% GPs and all urologists always or often informed on PSA testing during an early detection of cancer examination. In case of discomfort in the lower urinary tract, 78.5% urologists showed a more proactive approach of informing men on PSA testing vs. 41.5% GPs and in case of a positive family anamnesis (92.9% urologists vs. 75.7% GPs). 53.7% GPs replied that the proportion of men aged 45 years and older that finally receives (at least) one PSA test is almost none vs. 78.5% urologists. 57.1% urologists chose 10–14 years of life expectancy for an asymptomatic patient to recommend a PSA test vs. 39% GPs which would not recommend a test at all.

#### 3.5.1 Knowledge of PSA, risk factors and available guidelines

Clinicians’ knowledge about risk factors and PSA test characteristics was covered in 4 articles (22, 24, 30, 31). In addition, 4 studies focused on clinicians’ knowledge about the use of clinical practice guidelines (23, 26, 28, 29).

- Clinicians’ knowledge about risk factors and PSA test characteristics: Studies involving GPs showed a generally low level of awareness of the recommended uses of the test. In Malaysia (22), only 31% of respondents knew that having a first-degree relative with breast cancer was also a risk factor for PCa, and most of the GPs interviewed overestimated the predictive value of the test. A study carried out in Spain (24) showed that clinicians who had a greater knowledge of PSA tended to request testing in older patients and more frequently questioned the usefulness of the test. In South Africa (30), only 5.1% of the primary health care provider workers had good knowledge about PCa and medical officers or GPs had better knowledge compared with other professionals. Urologists (31) showed a greater knowledge risk factors associated to PCa than GPs.
- Clinicians’ knowledge about the use of clinical practice guidelines: In relation to clinicians’ knowledge regarding clinical practice guidelines, in a study carried out among GPs in the Netherlands (28), only half of the interviewees stated that they were aware of the available recommendations, but these GPs followed them. In another study among GPs in Spain, although they were not aware of clinical practice guidelines, they stated that they would like to have more information related to PSA testing (26). In another study carried out by GPs in a hospital in the USA (23), 90% of those interviewed were familiar with the existing guidelines, although they did not follow them in routine practice.

#### 3.5.2 Beliefs regarding the usefulness, benefits, and risks of the test

Eight of the included studies (22–26, 28, 30, 32) assessed clinicians’ opinions about the usefulness of PSA, and three of them included an evaluation of its benefits and risks (21, 23, 32).

- Clinicians’ opinions about the usefulness of PSA: In general, GPs’ opinion of PSA was unfavourable. In a study (25) conducted in the USA, only 40% of GPs acknowledged that the test was a useful screening test. In another study carried out in the Netherlands (28), more than 60% of the GPs interviewed indicated that they would probably not recommend the test to their relatives. In a study in Spain (24), more than 60% of clinicians questioned the usefulness of the biomarker, and nearly 30% of them did not consider it useful for diagnosing PCa. In another study performed in South Africa (30), 40.7 of the surveyed primary health care provider workers had a negative attitude towards PCa screening, and this percentage was higher in nurses and community health workers compared with other professionals.
- Clinicians’ evaluation of PSA benefits and risks: In other studies (21, 23), GPs indicated that the risks related to PSA outweighed the benefits.

Urologists showed a positive opinion about PCa screening with PSA, mainly those whose patients were Afro-Caribbeans (32) with a higher risk of PCa.

#### 3.5.3 Use of PSA in routine practice

Six studies (22, 23, 28–30, 32) addressed the issue of shared decision-making with the patient, and eight (11, 22, 24–27, 29, 31, 33) evaluated how clinicians used PSA in routine practice.

- SDM with patient: In primary care, 50% of surveyed GPs engaged in SDM for PCa. In a study carried out in the USA (23), more than 50% of surveyed GPs carried out shared decision-making with the patient but only 24% felt comfortable discussing the risks and benefits of PSA with patients. Similarly, in another study (22), 61.2% of GPs discussed the implication of an abnormal result, but only 20.4% discussed treatment for PCa before PSA testing. In a study carried out in the Netherlands (28), less than 50% of surveyed GPs would offer detailed advice before ordering a PSA test to an asymptomatic man who asked for it. In the USA (29), GPs performed SDM in 50.4% of white men and 54.8% black me, and in South Africa (30) 40% of GPs had poor practice regarding SDM in PCa screening.

A similar percentage was seen in urologists (32), who 50% discussed pros and contras of PSA screening with patients.

- Use of PSA in practice: In general, screening was recommended in primary care for patients with risk factors for PCa. One of the studies carried out in the USA (25) showed that most GPs only recommended the test considering individual risk, and a smaller percentage of them never offered it. Similarly, in the Saudi Arabian study (27), only 2.8% of GPs did not routinely recommend PSA. In a study carried out in the Netherlands (28), most GPs only recommended screening in patients with risk factors. In contrast, in other studies, GPs did not take risk factors into account when recommending the test: a study in the USA (29) involving GPs indicated that only 29% of them informed their black patients of the risks involved and tended not to have shared decision-making discussions. Several studies showed that GPs disagreed with the age recommendations for PSA testing. In the Saudi Arabian study (27) approximately 60% of GPs recommended screening in patients over 80 years old. In a Spanish study (26), 75% of GPs disagreed with the age range at which the test was offered and most of them thought that the most appropriate time interval for requesting a new test was annually.

A study comparing practices between GPs and urologists in Germany (11) showed a more proactive practice among urologists; 75% of GPs and 100% of urologists informed patients om PSA testing during an early detection of cancer examination. Urologists were in favor of starting screening at an earlier age if the patient had a family history (31). In addition, since being Afro-Caribbean was an important factor to consider, some urologists performed PSA at an early age (40 years) and up to 75 years (32) in these patients. Urologists indicated that existing guidelines were not adapted to Afro-Caribbean patients.

## 4 Discussion

The review’s main findings reveal that GPs exhibited a lower level of knowledge concerning PCa risk factors and clinical practice guidelines for PSA usage than urologists. This knowledge gap contributes to GPs being less inclined to adhere to available recommendations. In addition, there were differences in opinion on the usefulness of the PSA test. Volk et al. (34) noted that medical specialty was a variable related to the probability of screening, with GPs more likely to use PSA test than internal medicine clinicians. This discrepancy may be attributed to the practice setting, as some clinicians, such as urologists, typically work in inpatient settings where preventive care is less implemented. In addition, only near 50% of GPs and urologists carried out SDM with patients for PCa screening with PSA test.

Most studies included in this review indicated that the main reason for GPs to screen patients with recognized PCa risk factors was their knowledge of these factors. However, studies involving GPs consistently showed a generally lower awareness of recommended PSA test applications and the associated PCa risk factors than urologists.

Most of the surveyed clinicians, aligned with previous studies conducted in Ireland and the USA (35), knew that having a relative with PCa and being older than 50 years old were risk factors for PCa. However, a low percentage of clinicians knew that having a first-degree relative with breast cancer increased the risk for PCa, even though in 2020, the EAU incorporated the recommendation to offer early PSA testing to well-informed men aged over 40 with BRCA2 mutations. In line with previous studies (36), a significant finding in this review was that more than 50% of surveyed GPs tended to overestimate the positive predictive value of PSA (22), possibly indicating a lack of awareness among clinicians, potentially resulting in excessive screening and inadequate information provided to patients about the PSA test.

Some studies reported disagreement among GPs regarding the age range at which the test should be offered (26). In contrast, urologists, demonstrated a more proactive stance (11) expressing a willingness to initiate screening at an earlier age for patients with a family history of PCa (31). A previous study carried out in the United States found no differences between urologists and primary care clinicians in the number of PSA tests carried out (37), which could be explained by the differences in health care systems.

Clinicians’ awareness of clinical practice guidelines was generally low, and even those who were aware did not consistently follow them in routine practice (23). Previous research also showed that clinicians generally had favorable attitudes toward clinical guidelines, but that only one-third used them very often or often (38). This lack of adherence could be attributed to the constant updates and lack of consensus in guidelines, which lead to confusion (26). Some GPs, although unaware of clinical practice guidelines, expressed a desire for more information related to PSA testing (26). Urologists, on the other hand, argued that existing guidelines were not adapted to high-risk patients, such as Afro-Caribbean patients (32).

Previous studies indicated that clinicians’ insufficient knowledge was linked to variation in PSA testing practices (39, 40). This systematic review further revealed that clinicians with lower PSA knowledge tended to request testing in younger patients and were less likely to question the test’s usefulness. Overall, GPs exhibited an unfavorable opinion of PSA, half of them stating that they would probably not recommend the test to their relatives, emphasizing perceived risks outweighing benefits. In contrast, urologists generally held a positive opinion of PCa screening with PSA, especially for patients at higher risk of PCa.

Less than half of the included studies assessed the implementation of SPM in practice. Both GPs and urologists were found to involve patients in SPM on PCa screening at a rate of 50% or less, highlighting a potential gap in this context. The lack of shared decision-making when ordering PSA screening is of relevant concern. Evidence suggests that clinicians have traditionally underestimated the adverse impact of PSA determination (11), and consequently, it is rarely explained to patients (41), although several studies show that most wish to be informed (26). In this review, it was shown that GPs rarely discussed PSA screening with their patients, although it is critical to help them to make informed decisions regarding screening. Major professional organizations have strongly recommended that patients be fully informed about the pros, cons, and uncertainties of PSA screening, enabling them to make a decision based on their specific clinical and personal characteristics (42). Information regarding test properties such as the likelihood of having a false-positive result or overdiagnosis are not frequently explained to patients (43) and should be explained in the context of their characteristics.

The findings of this systematic review shed some light on the complexity of decision making in oncology, which leads patients and clinicians to consider the benefits and risks of an increasing number of clinical options. Patients and clinicians evaluate the options differently, and therefore, all relevant information and personal preferences are needed to make a decision. This review has also shown that a clinician’s personal beliefs and specialization can influence the use of PSA testing (32), leading to significant variability in practice. These results are consistent with those observed prior to the guideline update, indicating that physicians’ knowledge, beliefs and practices regarding PSA testing have not been influenced by the available recommendations. Hence, and in accordance with the recent recommendations from the European Association of Urology (EAU) (6), clinicians should receive more training in PSA testing.

The overarching strength of this study was the comparison between urologists and GPs given the different roles of these clinicians in opportunistic PCa screening. In addition, the different analyses of the clinicians’ knowledge, beliefs and practice allow us to evaluate different aspects that may influence the recommendation for opportunistic PCa screening.

This systematic review has, however, some limitations. The review was restricted by some terms, e.g., and some relevant studies may not have been included. However, in relation to the language limitation, although we restricted the search to English and Spanish, we also checked for publication in other languages and there were no studies that met the eligibility criteria. This review did not identify studies that evaluated clinicians’ knowledge on the use of genetic biomarkers together with PSA detection for PCa screening. However, the use of additional tests, such as certain genes or molecules shed into urine—TMPTSS2:ERG gene fusions or PCA3 mRNA—has been suggested as a way to reduce overdiagnosis (33). However, GPs usually considered the presence of patient risk factors such as race, age or family history when ordering a PSA, although they were less in favour of carrying out PSA determination than urologists. Thus, they could also support the inclusion of other risk factors, such as genetic risk stratification, which will allow them to advance toward personalized management of the patient. We did not register the protocol of this systematic review in PROSPERO (44). Registration minimizes unintentional duplication of systematic reviews and enhance transparency in the review process, thereby mitigating reporting bias. However, this systematic review conforms to reporting Guidelines PRISMA and a protocol has also included. The adherence to the 22 STROBE criteria demonstrated a high median compliance, with only three studies falling below the first quartile. Most unmet criteria were associated with conducting sensitivity analyses, reporting the estimation of the sample size, and handling quantitative variables. Importantly, these criteria are not directly linked to information bias in the selection process, suggesting that their omission may have minimal impact on the precision of the results.

## 5 Conclusion

From the findings of this review and considering the new recommendations published by the European Commission and Urologist Associations (5–8), we identify the need to carry out education interventions with trusted resources based on the available evidence and the current guidelines, mainly in the implementation in practice of SDM. This knowledge will allow health professionals to develop shared decision-making with patients when ordering a PSA.

## Data availability statement

The original contributions presented in the study are included in the article/Supplementary material, further inquiries can be directed to the corresponding author.

## Author contributions

ME-O: Conceptualization, Data curation, Formal analysis, Methodology, Writing – original draft. CC: Data curation, Methodology, Writing – review & editing. AM-L: Data curation, Methodology, Writing – review & editing. LP: Data curation, Formal analysis, Methodology, Writing – review & editing. JC-R: Data curation, Formal analysis, Methodology, Writing – review & editing. BL: Conceptualization, Data curation, Formal analysis, Funding acquisition, Methodology, Validation, Writing – original draft, Writing – review & editing.
