# Supplementary material for: Urologists’ and general practitioners’ knowledge, beliefs and practice relevant for opportunistic prostate cancer screening: a PRISMA-compliant systematic review
Source: Front Med (Lausanne). 2024 Feb 16;11:1283654. doi: 10.3389/fmed.2024.1283654 (PMC10905619; doi:10.3389/fmed.2024.1283654)
Supplement: Supplementary file 2 [file Table_2.docx]

Supplementary Material

**Supplementary Table 1: Protocol of the study**

| **Review title** | Urologists’ and general practitioners’ knowledge, beliefs and practice relevant for opportunistic prostate cancer screening: a PRISMA-compliant systematic review |
| --- | --- |
| **Organisational affiliation of the review** | Universidad Miguel Hernandez |
| **Review team members and their organisational affiliations** | - Blanca Lumbreras. Universidad Miguel Hernandez - María Estevan-Ortega. Universidad Miguel Hernandez - Cristina de la Encarnación Castellano. Hospital General de Alicante - Alberto Mendiola López. Hospital General de Alicante - Juan Pablo Caballero. Hospital General de Alicante - Lucy A Parker. Universidad Miguel Hernandez |
| **Funding sources/sponsors** | Instituto de Salud Carlos III (PI20/01334) |
| **Review question** | The aim of this systematic review is to analyze the recent evidence of clinicians’ knowledge (urologists  and general practitioners) and practice regarding opportunistic prostate cancer screening with PSA  determination. |
| **Searches** | MEDLINE (through PubMed), Web of Science and EMBASE |
| **Search strategy** | ("Prostatic Neoplasms" [MeSH Terms] OR "Mass Screening" [MeSH Terms] OR "prostatic neoplasms/diagnosis" [MeSH Terms] OR "prostatic neoplasms/prevention and control" [MeSH Terms]) AND ("Diagnostic Screening Programs"[Mesh] OR "Early Detection of Cancer"[Mesh] OR "Prostate-Specific Antigen"[Mesh] OR "prostate-specific antigen (154-163)" [Supplementary Concept] OR "prostate-specific antigen (146-154)" [Supplementary Concept])) AND ("Urologists" [MeSH Terms] OR "Professional Review Organizations" [MeSH Terms] OR "physicians, family" [MeSH Terms] OR "physicians, primary care" [MeSH Terms] OR "General Practitioners" [MeSH Terms] OR "Physicians" [MeSH Terms]) AND ((health knowledge, attitudes, practice" [MeSH Terms] OR "Medical Informatics" [MeSH Terms] OR "Information Dissemination" [MeSH Terms] OR "Decision Trees" [MeSH Terms] OR "education, medical" [MeSH Terms] OR "Knowledge" [MeSH Terms] OR "physicians/education" [MeSH Terms] OR "Diagnostic Techniques and Procedures" [MeSH Terms] OR "Access to Information" [MeSH Terms] OR "Surveys and Questionnaires" [MeSH] |
| **Condition or domain being studied** | Opportunistic screening for Prostate Cancer with PSA should be carry out after the patient has understood the benefits and risks of screening. Available guidelines have been recently updated and there is no evidence about clinicians' knowledge, attitudes and practices on opportunistic screening of prostate cancer with PSA test. |
| **Participants/population** | General practitioners and urologists |
| **Intervention(s), exposure(s)** | Opportunistic screening of prostate cancer based on PSA test. |
| **Types of study to be included** | Observational studies |
| **Main outcome(s)** | Clinicians’ knowledge (urologists and GPs), attitudes and practice regarding opportunistic prostate cancer  screening with PSA determination. |
| **Data extraction** | Study selection: In the first stage of study selection, two review authors independently will screen the titles and abstracts of the retrieved records, eliminating duplicates and studies that were clearly ineligible. We then will retriev and read the full-text articles of the remaining records, selecting those that met our inclusion criteria. All discrepancies will be resolved through discussion with the rest of the review team. |
| **Risk of bias (quality) assessment** | Studies will be assessed for reporting quality according to the STrengthening the Reporting of Observational studies in Epidemiology (STROBE) guidelines. Three of the authors will review the studies independently, and disagreements will be resolved by discussion and consensus with other reviewer. |
| **Strategy for data synthesis** | Data will be collated and synthesised using narrative and descriptive summaries. No attempt at meta-analysis will be made given the heterogeneity in target population, study design and outcome measures across included studies. To improve conceptual clarity and comprehensiveness, two independent researchers will synthesize for each report the knowledge, attitudes and practice for the different population (i.e., general practitioners, urologists). |
| **Analysis of subgroups or subsets** | Results will be analysed by participant characteristics: urologists and general practitioners. |
| **Keywords** | Prostate cancer; screening; knowledge; beliefs; practice; urologists; general practitioners. |

**Supplementary Table 2:** Searches for descriptors are carried out in English and combined by Boolean operators (OR and AND) in four blocks: clinicians; prostate cancer; screening; knowledge, attitudes, practices. The descriptors in each block are combined by the Boolean operator OR. The combination between the blocks is done using the AND operator.

| clinicians | prostate cancer | screening | knowledge, beliefs, practices |
| --- | --- | --- | --- |
| “Urologists [MeSH Terms]  OR "Professional Review Organizations" [MeSH Terms]  OR "physicians, family" [MeSH Terms]  OR "physicians, primary care" [MeSH Terms]  OR "General Practitioners" [MeSH Terms]  OR "Physicians" [MeSH Terms] | "Prostatic Neoplasms" [MeSH Terms]  OR "prostatic neoplasms/diagnosis" [MeSH Terms]  OR "prostatic neoplasms/prevention and control" [MeSH Terms] | "Diagnostic Screening Programs"[Mesh]  OR "Early Detection of Cancer"[Mesh]  OR "Prostate-Specific Antigen"[Mesh]  OR "prostate-specific antigen (154-163)" [Supplementary Concept]  OR "prostate-specific antigen (146-154)" [Supplementary Concept]) | ““health knowledge, attitudes, practice" [MeSH Terms]  OR "Medical Informatics" [MeSH Terms]  OR "Information Dissemination" [MeSH Terms]  OR "Decision Trees" [MeSH Terms] OR "education, medical" [MeSH Terms]  OR "Knowledge" [MeSH Terms]  OR "physicians/education" [MeSH Terms]  OR "Diagnostic Techniques and Procedures" [MeSH Terms]  OR "Access to Information" [MeSH Terms]  OR "Surveys and Questionnaires" [MeSH]) |

**Supplementary Table 3: Description of the compliance with items included in the checklist of the STrengthening the Reporting of OBservational studies in Epidemiology (STROBE) guidelines.**

| Item from STROBE Checklist | | | Compliance | |
| --- | --- | --- | --- | --- |
|  |  |  | **N** | **(%)** |
| Title and abstract |  |  |  |  |
|  | 1 | Indicate the study’s design with a commonly used term in the title or the abstract  Provide in the abstract an informative and balanced summary of what was done and what was found | 10 | 71 |
| Introduction |  |  |  |  |
| Background/rationale | 2 | Explain the scientific background and rationale for the investigation being reported | 14 | 100 |
| Objectives | 3 | State specific objectives, including any prespecified hypotheses | 12 | 86 |
| Methods |  |  |  |  |
| Study design | 4 | Present key elements of study design early in the manuscript | 14 | 100 |
| Setting | 5 | Describe the setting, locations, and relevant dates, including periods of recruitment, exposure, follow-up, and data collection | 11 | 79 |
| Participants | 6 | Cohort study - give the eligibility criteria, and the sources and methods of selection of participants; describe methods of follow-up  Case-control study - give the eligibility criteria, and the sources and methods of case ascertainment and control selection; give the rationale for the choice of cases and controls  Cross-sectional study - give the eligibility criteria, and the sources and methods of selection of participants  Cohort study - for matched studies, give matching criteria and number of exposed and unexposed Case-control study - for matched studies, give matching criteria and the number of controls per case | 14 | 100 |
| Variables | 7 | Clearly define all outcomes, exposures, predictors, potential confounders, and effect modifiers; give diagnostic criteria, if applicable | 12 | 86 |
| Data sources/measurement | 8* | For each variable of interest, give sources of data and details of methods of assessment (measurement); describe comparability of assessment methods if there is more than one group | 10 | 71 |
| Bias | 9 | Describe any efforts to address potential sources of bias | 8 | 57 |
| Study size | 10 | Explain how the study size was arrived at | 8 | 57 |
| Quantitative variables | 11 | Explain how quantitative variables were handled in the analyses; if applicable, describe which groupings were chosen and why | 14 | 100 |
| Statistical methods | 12 | Describe all statistical methods, including those used to control for confounding  Describe any methods used to examine subgroups and interactions  Explain how missing data were addressed  Cohort study - if applicable, explain how loss to follow-up was addressed  Case-control study - if applicable, explain how matching of cases and controls was addressed  Cross-sectional study - if applicable, describe analytical methods taking account of sampling strategy  Describe any sensitivity analyses | 10 | 71 |
| Results |  |  |  |  |
| Participants | 13* | Report numbers of individuals at each stage of study - e.g., numbers potentially eligible, examined for eligibility, confirmed eligible, included in the study, completing follow-up, and analyzed  Give reasons for nonparticipation at each stage Consider use of a flow diagram | 10 | 71 |
| Descriptive data | 14* | Give characteristics of study participants (e.g., demographic, clinical, social) and information on exposures and potential confounders  Indicate number of participants with missing data for each variable of interest Cohort study - summarize follow-up time (e.g., average and total amount) | 14 | 100 |
| Outcome data | 15* | Cohort study - report numbers of outcome events or summary measures over time  Case-control study - report numbers in each exposure category, or summary measures of exposure  Cross-sectional study - report numbers of outcome events or summary measures | 14 | 100 |
| Main results | 16 | Give unadjusted estimates and, if applicable, confounder-adjusted estimates and their precision (e.g., 95% confidence interval); make clear which confounders were adjusted for and why they were included Report category boundaries when continuous variables were categorized  If relevant, consider translating estimates of relative risk into absolute risk for a meaningful time period | 9 | 64 |
| Other analyses | 17 | Report other analyses done - e.g., analyses of subgroups and interactions, and sensitivity analyses | 4 | 29 |
| Discussion |  |  |  |  |
| Key results | 18 | Summarize key results with reference to study objectives | 14 | 100 |
| Limitations | 19 | Discuss limitations of the study, taking into account sources of potential bias or imprecision; discuss both direction and magnitude of any potential bias | 14 | 100 |
| Interpretation | 20 | Give a cautious overall interpretation of results considering objectives, limitations, multiplicity of analyses, results from similar studies, and other relevant evidence | 14 | 100 |
| Generalizability | 21 | Discuss the generalizability (external validity) of the study results | 13 | 93 |
| Other information |  |  |  |  |
| Funding | 22 | Give the source of funding and the role of the funders for the present study and, if applicable, for the original study on which the present article is based | 8 | 57 |

**Supplementary Table 4: Description of the compliance with items included in the checklist of the STrengthening the Reporting of OBservational studies in Epidemiology (STROBE) guidelines for each individual study included in the systematic review.**

| **Study** | **Item from STROBE Checklist** | | | | | | | | | | | | | | | | | | | | | | |
| --- | --- | --- | --- | --- | --- | --- | --- | --- | --- | --- | --- | --- | --- | --- | --- | --- | --- | --- | --- | --- | --- | --- | --- |
|  | **1** | **2** | **3** | **4** | **5** | **6** | **7** | **8** | **9** | **10** | **11** | **12** | **13** | **14** | **15** | **16** | **17** | **18** | **19** | **20** | **21** | **22** | **Total** |
| Elstad EA, 2015 (21) | Yes | Yes | Yes | Yes | No | Yes | Yes | Yes | Yes | No | Yes | Yes | Yes | Yes | Yes | Yes | No | Yes | Yes | Yes | Yes | No | **18** |
| Malik A, 2016 (22) | Yes | Yes | Yes | Yes | Yes | Yes | Yes | Yes | No | Yes | Yes | Yes | Yes | Yes | Yes | Yes | No | Yes | Yes | Yes | Yes | Yes | **20** |
| Miller A, 2016 (23) | No | Yes | Yes | Yes | No | Yes | Yes | No | No | No | Yes | No | Yes | Yes | Yes | No | No | Yes | Yes | Yes | Yes | No | **13** |
| Panach-Navarrete J, 2016 (24) | Yes | Yes | Yes | Yes | No | Yes | No | No | No | Yes | Yes | Yes | Yes | Yes | Yes | Yes | No | Yes | Yes | Yes | Yes | Yes | **17** |
| Hall IJ, 2017 (25) | No | Yes | No | Yes | Yes | Yes | Yes | Yes | Yes | Yes | Yes | Yes | Yes | Yes | Yes | Yes | Yes | Yes | Yes | Yes | Yes | Yes | **18** |
| Giménez N, 2018 (26) | Yes | Yes | Yes | Yes | Yes | Yes | Yes | Yes | Yes | Yes | Yes | Yes | Yes | Yes | Yes | Yes | Yes | Yes | Yes | Yes | Yes | No | **20** |
| Nassir AM, 2019 (27) | Yes | Yes | Yes | Yes | Yes | Yes | Yes | Yes | Yes | Yes | Yes | Yes | Yes | Yes | Yes | Yes | Yes | Yes | Yes | Yes | Yes | Yes | **22** |
| Kappen S, 2020 (28) | Yes | Yes | Yes | Yes | Yes | Yes | Yes | Yes | Yes | Yes | Yes | Yes | Yes | Yes | Yes | Yes | Yes | Yes | Yes | Yes | Yes | Yes | **21** |
| Shungu N, 2022 (29) | Yes | Yes | Yes | Yes | Yes | Yes | Yes | Yes | Yes | No | Yes | Yes | Yes | Yes | Yes | Yes | No | Yes | Yes | Yes | Yes | No | **19** |
| Benedict MOA, (30) | No | Yes | Yes | Yes | Yes | Yes | Yes | No | Yes | Yes | Yes | No | Yes | Yes | Yes | No | No | No | Yes | Yes | Yes | Yes | **16** |
| Rudichuk L, 2017 (31) | Yes | Yes | Yes | Yes | Yes | Yes | No | No | Yes | Yes | Yes | Yes | Yes | Yes | Yes | Yes | No | Yes | Yes | Yes | Yes | Yes | **17** |
| Persaud S, 2018 (32) | Yes | Yes | Yes | Yes | Yes | Yes | Yes | Yes | No | No | Yes | No | Yes | Yes | Yes | No | No | Yes | Yes | Yes | Yes | Yes | **17** |
| Scherer TM, 2023 (33) | Yes | Yes | Yes | Yes | Yes | Yes | Yes | Yes | No | No | Yes | Yes | Yes | Yes | Yes | No | No | Yes | Yes | Yes | No | Yes | **17** |
| Kappen S, 2019 (11) | No | Yes | No | Yes | Yes | Yes | Yes | Yes | No | No | Yes | No | Yes | Yes | Yes | No | No | Yes | Yes | Yes | Yes | Yes | **15** |
| Total | 10 | 14 | 12 | 14 | 11 | 14 | 12 | 10 | 8 | 8 | 14 | 10 | 14 | 14 | 14 | 11 | 4 | 13 | 14 | 14 | 13 | 10 |  |
